# 2760. Fecal Microbiota Transplantation for Decolonization of Carbapenemase-Producing Enterobacterales

**DOI:** 10.1093/ofid/ofad500.2371

**Published:** 2023-11-27

**Authors:** Benjamin Davido, Andrea Watson, Pierre De Truchis, Gianluca Galazzo, Aurelien Dinh, Rui Batista, Elisabeth M Teerver, Christine Lawrence, Hugues Michelon, Marion Jobard, Ed J Kuijper, Silvia Caballero

**Affiliations:** Raymond Poincaré Teaching Hospital, Garches, Ile-de- France, France; Vedanta Biosciences, Cambridge, Massachusetts; Hopital Raymond Poincaré, Garches, Ile-de- France, France; Vedanta Biosciences, Cambridge, Massachusetts; Raymond Poincaré University Hospital, Paris, Ile-de- France, France; Hopital Cochin, Paris, Ile-de- France, France; Leiden University Medical Centre, Leiden, Utrecht, Netherlands; Hôpital Raymond-Poincaré, Garches, Ile-de- France, France; Hopital Raymond Poincaré, Garches, Ile-de- France, France; Hopital Cochin, Paris, Ile-de- France, France; Leiden University Medical Center and RIVM, Leiden, Zuid-Holland, Netherlands; Vedanta Biosciences, Cambridge, Massachusetts

## Abstract

**Background:**

Fecal microbiota transplantation (FMT) can effectively decolonize multidrug-resistant organisms but its efficacy is variable. We evaluated the impact of FMT treatment at eliminating carbapenemase-producing Enterobacterales (CPE) carriage compared to spontaneous decolonization and identified microbial signatures of successful decolonization.

**Methods:**

Prospective study, with patients colonized with CPE receiving a single dose of FMT via the nasogastric route. CPE-colonized patients who did not meet our inclusion criteria or decided not to participate in the study comprised the control group where spontaneous decolonization was evaluated. Primary endpoint was complete elimination of CPE carriage 2 weeks after FMT. A secondary endpoint for decolonization was set at 3 months post-FMT. Successful decolonization was defined by 2+ consecutive negative rectal swabs as determined by both culture and PCR. Shotgun metagenomic sequencing was performed to assess gut microbiota composition of donors and recipients before and after FMT.

**Results:**

Twenty patients colonized with CPE were included, 72.5 ± 18.8 years. Median duration of carriage of CPE before FMT was 62.5 days (IQR 48.75-122.5). At 3 months post-FMT, 40% (n=8/20) of patients were successfully decolonized, while 20% (n=4/20) meeting the 2-week primary endpoint. Kaplan-Meier curves between patients in the FMT cohort and those in the control group (n=82) revealed the same rate of decolonization over time (p=0.9). Microbiota composition analyses post-FMT revealed significantly higher bacterial species richness and alpha diversity in responders versus non-responders (Fig1A,B), and specific taxa including *Faecalibacterium prausnitzii*, *Parabacteroides distasonis*, *Collinsella aerofaciens*, *Alistipes finegoldii* and *Blautia_A sp900066335* were associated with response (Fig.2). Furthermore, CPE abundance levels at baseline were significantly higher in non-responders than in responders (Fig.3).

**Figure:**
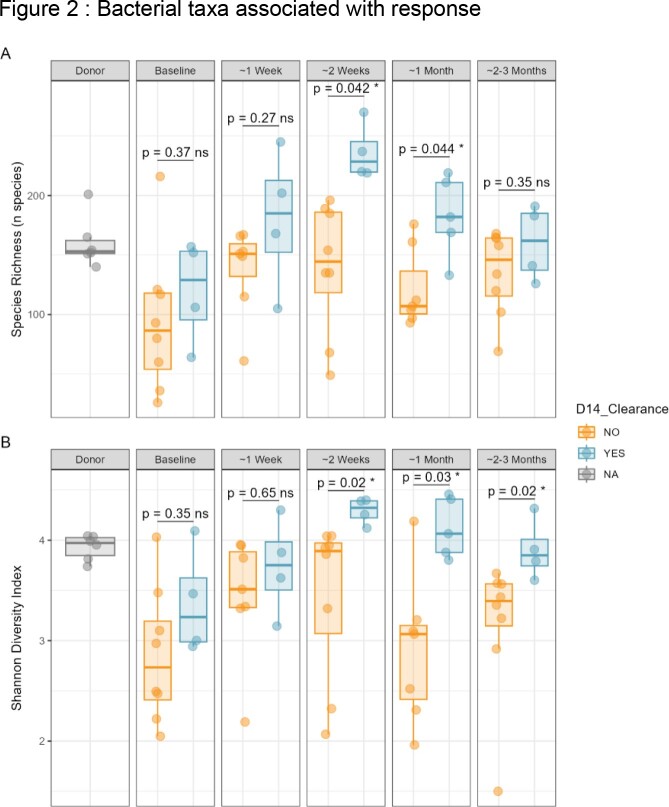

Box and whisker plots of the percent relative abundance for the indicated species in samples from patients with CRE clearance on day 14 (teal), and patients without CRE clearance on day 14 (orange). The median (±interquartile range) is plotted for each species in each group. Whiskers extend to the lowest and highest values no greater than 1.5x the interquartile range from the closest hinge. The displayed taxa were significantly altered between patients with and without CRE clearance at day 14 across all samples, with q-values below 0.01 (MaAslin2 log-transformed linear model).

**Figure:**
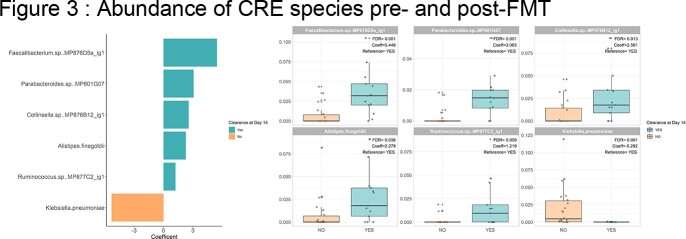

Box and whisker plots indicating the cumulative percent relative abundance of CRE species in samples from patients with CRE clearance on day 14 (teal) and patients without CRE clearance on day 14 (orange). The median (±interquartile range) is plotted for each group at the indicated timepoints. Whiskers extend to the lowest and highest values no greater than 1.5x the interquartile range from the closest hinge. Patients with CRE clearance on day 14 have significantly lower CRE relative abundance than patients without CRE clearance on day 14 at baseline and 1 week post-FMT (Wilcoxen Rank-Sum test with FDR adjustment).

**Figure:**
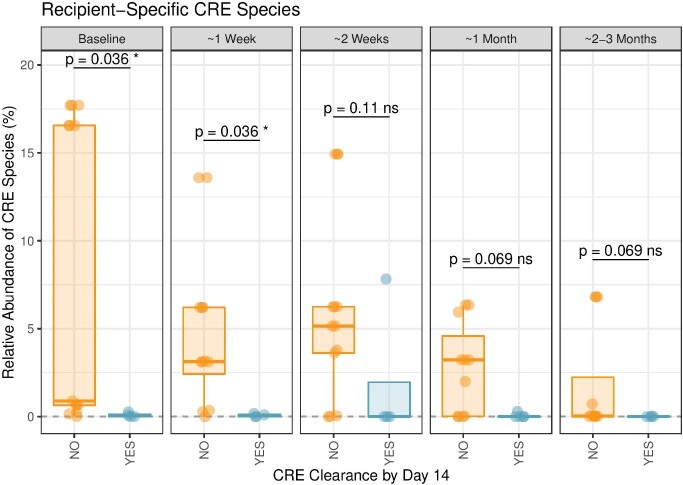

**Conclusion:**

FMT administration led to complete CPE eradication in a subset of colonized patients but did not achieve statistical significance. Response to FMT was not donor-dependent and correlated with the transfer of specific bacteria taxa and CPE colonization levels.

**Disclosures:**

**Andrea Watson, PhD**, Vedanta Biosciences: Advisor/Consultant|Vedanta Biosciences: Honoraria **Gianluca Galazzo, PhD**, Vedanta Biosciences: Advisor/Consultant|Vedanta Biosciences: Honoraria **Ed J. Kuijper, Prof. Dr.**, Vedanta Biosciences: Advisor/Consultant **Silvia Caballero, PhD**, Vedanta Biosciences: Advisor/Consultant|Vedanta Biosciences: Board Member|Vedanta Biosciences: Ownership Interest

